# Deriving detector‐specific correction factors for rectangular small fields using a scintillator detector

**DOI:** 10.1120/jacmp.v17i6.6433

**Published:** 2016-11-08

**Authors:** Yujiao Qin, Hualiang Zhong, Ning Wen, Karen Snyder, Yimei Huang, Indrin J. Chetty

**Affiliations:** ^1^ Department of Radiation Oncology Henry Ford Health System Detroit MI USA

**Keywords:** small field, plastic scintillator detector, collimator exchange, output factor

## Abstract

The goal of this study was to investigate small field output factors (OFs) for flattening filter‐free (FFF) beams on a dedicated stereotactic linear accelerator‐based system. From this data, the collimator exchange effect was quantified, and detector‐specific correction factors were generated. Output factors for 16 jaw‐collimated small fields (from 0.5 to 2 cm) were measured using five different detectors including an ion chamber (CC01), a stereotactic field diode (SFD), a diode detector (Edge), Gafchromic film (EBT3), and a plastic scintillator detector (PSD, W1). Chamber, diodes, and PSD measurements were performed in a Wellhofer water tank, while films were irradiated in solid water at 100 cm source‐to‐surface distance and 10 cm depth. The collimator exchange effect was quantified for rectangular fields. Monte Carlo (MC) simulations of the measured configurations were also performed using the EGSnrc/DOSXYZnrc code. Output factors measured by the PSD and verified against film and MC calculations were chosen as the benchmark measurements. Compared with plastic scintillator detector (PSD), the small volume ion chamber (CC01) underestimated output factors by an average of ‐1.0%±4.9%(max.=‐11.7% for $0.5\times 0.5\;{\text{cm}}^{2}$ square field). The stereotactic diode (SFD) overestimated output factors by 2.5%±0.4%(max.=3.3% for $0.5\times 1\;{\text{cm}}^{2}$ rectangular field). The other diode detector (Edge) also overestimated the OFs by an average of 4.2%±0.9%(max.=6.0% for $1\times 1\;{\text{cm}}^{2}$ square field). Gafchromic film (EBT3) measurements and MC calculations agreed with the scintillator detector measurements within 0.6%±1.8% and 1.2%±1.5%, respectively. Across all the X and Y jaw combinations, the average collimator exchange effect was computed: 1.4%±1.1% (CC01), 5.8%±5.4% (SFD), 5.1%±4.8% (Edge diode), 3.5%±5.0% (Monte Carlo), 3.8%±4.7% (film), and 5.5%±5.1% (PSD). Small field detectors should be used with caution with a clear understanding of their behaviors, especially for FFF beams and small, elongated fields. The scintillator detector exhibited good agreement against Gafchromic film measurements and MC simulations over the range of field sizes studied. The collimator exchange effect was found to be important at these small field sizes. Detector‐specific correction factors were computed using the scintillator measurements as the benchmark.

PACS number(s): 87.56.Fc

## I. INTRODUCTION

The concept of small field dosimetry has been introduced since the implementation of stereotactic radiosurgery.^1^, ^2^ With the recent developments in frameless radiosurgery and intensity‐modulated radiotherapy (IMRT), a wider discussion on the topic has been triggered within the medical physics community.^3^, ^4^, ^5^, ^6^, ^7^ Small field measurements require the use of appropriate detectors, and must be performed with utmost attention to the setup accuracy. These measurements are challenging, and are fundamentally linked to the physics of small fields. Unlike traditional large fields, electronic equilibrium breaks down as the radiation field size drops below the range of the secondary electrons, invalidating the Bragg‐Gray conditions.^8^, ^9^ Source occlusion also takes place at small fields, resulting in a decrease in the dose deposited.^(4)^ In addition, measurements at small fields are limited by the various detector‐specific effects.^4^, ^9^, ^10^ These include the volume averaging around high‐gradient dose distributions, the fluence/dose perturbations introduced by the different physical densities between detector and medium, and the uncertainty in detector geometric alignment.

For dosimetry in nonreference conditions such as small fields and composite IMRT fields, the International Atomic Energy Agency (IAEA) and the American Association of Physicist in Medicine (AAPM) have recommended a new formalism^(10)^ as an extension to the existing Task Group 51.^(11)^ An additional correction factor $k_{Q_{\text{clin},Q_{\text{msr}}}}^{f_{\text{clin},f_{\text{msr}}}}$ was introduced to account for the detector‐specific effects at these nonreference conditions. Mathematically, $k_{Q_{\text{clin},Q_{\text{msr}}}}^{f_{\text{clin},f_{\text{msr}}}}$ is the ratio of output factor obtained by the reference versus that measured by the detector of interest.^(10)^

Many studies have reported on the value of $k_{Q_{\text{clin},Q_{\text{msr}}}}^{f_{\text{clin},f_{\text{msr}}}}$ Among those, radiochromic film, plastic scintillator detector (PSD) and Monte Carlo simulation are often used to obtain reference doses at small fields. Regardless of which reference was chosen, each has its own caveat. Radiochromic film requires postprocessing and is prone to interbatch variability;^(23)^ scintillator detectors are susceptible to background Cherenkov radiation;^(24)^ the accuracy of Monte Carlo calculation depends heavily on the accuracy of the modeling of treatment head structures and the statistical uncertainty of the simulation.^(25)^ There is a paucity of studies in the literature reporting on the comparison of a scintillator detector, Gafchromic film measurements, and MC simulations for small field output factors (OFs). In this study, all the aforementioned methods were employed for estimating small field OFs; the scintillator detector was selected as the reference.

The collimator exchange effect has been reported for Elekta and Siemens machines at small rectangular field sizes.^(17)^ The collimator exchange effect describes the difference in OFs in rectangular field, depending on which side of the rectangle delineates the jaws.^(26)^ It results from the difference in X and Y jaw backscatter into the monitor chamber. Task Group 74 was one of the first to define this concept. Collimator exchange effect in the range of 3%–5% was reported for Elekta, Siemens, and Varian machines at three different energies.^(26)^ However, the smallest field size TG‐74 studied was 4 cm. With the recent improvement in image quality and localization accuracy, stereotactic radiosurgery (SRS) often target much smaller tumors. At small fields, the extent of Y jaw backscatter increases exponentially, contributing to a significant decrease in OF.^(26)^ Consequently, the collimator exchange effect should be quantified and accounted for in the treatment planning system for small fields. There is currently limited literature reported on the collimator exchange effect in small fields^(17)^ even fewer on flattening filter‐free (FFF) beam. As the amount of scattered photons was reduced in FFF beam, the extent of the collimator exchange effect may be different. Since most linac‐based SRS utilizes FFF beam, quantifying such effect would help us understand any quality assurance (QA) discrepancies observed between measurement and dose calculation, especially for small elongated target like spinal ependymoma.

Therefore, the purpose of this study was, first, to provide a comprehensive evaluation of small field OFs, using different detectors, for FFF beams from a dedicated stereotactic radiosurgery linac (Edge, Varian Medical Systems, Palo Alto, CA). We then quantified the small field collimator exchange effect for each detector. From this data, detector‐specific correction factors $k_{Q_{\text{clin},Q_{\text{msr}}}}^{f_{\text{clin},f_{\text{msr}}}}$ were derived.

## II. MATERIALS AND METHODS

The OF is defined as $D_{\text{FS}}/D_{10\times 10}$, the ratio of dose at given field size to the dose at $10\times 10\;{\text{cm}}^{2}$. OFs for 16 jaw‐based square and rectangular fields, with X and Y jaw combinations each set to 0.5, 1, 1.5, and 2 cm, were measured and simulated for a 6XFFF beam on the Varian Edge linac. Width‐to‐length (X to Y) ratio ranged from 0.25 to 4. MLCs were fully retracted.

Five different detectors were included in this study: CC01 ion chamber (IBA Dosimetry, Schwarzenbruck, Germany), stereotactic field diode (SFD; IBA Dosimetry), Edge detector (Sun Nuclear, Melbourne, FL), Exradin W1 scintillator detector (PSD; Standard Imaging, Middleton, WI), and Gafchromic EBT3 film (Ashland, Inc., Bridgewater, NJ). The characteristics of these detectors are listed in Table 1. The collimator exchange effect (CE) was quantified, as noted in Eq. (1):

$$CE(X,Y)=\frac{OF(X,Y)}{OF(Y,X)}-1$$

where *X* and *Y* were the respective X and Y jaw settings. For example, the CE of fields (0.5, 2 cm) is $[{\text{OF}}_{\left(0.5\times 2\text{cm}\right)}/{\text{OF}}_{\left(2\times 0.5\text{cm}\right)}]‐1$. The CEs were then compared among all detectors and against MC calculations. The correlation between width‐to‐length ratio and CE was calculated to investigate the impact of field elongation on OFs.

Based on the formalism proposed by Alfonso et al.,^(10)^ detector‐specific correction factors for all 16 fields were calculated using Eq. (2), considering the plastic scintillator detector (PSD) as the reference:

$$k_{Q_{clin},Q_{msr}}^{f_{clin},f_{msr}}=\frac{OF_{PSD}}{OF_{\text{detector}}}$$

**Table 1:** Detectors used for measurement of output factor

*Detector*	*Acronym*	*Type*	*Material*	*Active Volume (mm* ^*3*^ *)*	*Dimension*
Scanditronix CC01	CC01	thimble chamber	steel central electrode	10	inner radius 1 mm
Scanditronix SFD	SFD	p‐type diode	silicon	0.017	0.6 mm diameter, 0.06 mm length
Sun Nuclear Edge detector	Edge detector	n‐type diode	silicon	0.019	0.8 mm width, 0.03 mm thickness, 0.8 mm length
Exradin W1 Scintillator	PSD	plastic scintillator	polystyrene	2	1 mm diameter, 3 mm length
Gafchromic EBT3 film	Film	radiochromic film	photopolymer with marker dye	NA	top polyester 50 microns, active layer 30 microns, bottom polyester 175 microns

### A. Chamber, diodes, and PSD measurement

Percent depth doses (PDD), profiles, and output factors were obtained in a Wellhofer IBA Blue Water Phantom tank (IBA, Stockholm, Sweden) using a 3D scanning system. The 100 cm source‐to‐surface distance (SSD) was set to the water surface. OFs were measured using the CC01, SFD, PSD, and the Edge detector, with effective points of measurement for each detector located at 10 cm depth. PDDs and profiles at 10 cm depth were scanned with the Edge detector. The tank was leveled prior to dose delivery and vertical travel of the detectors was verified. The CC01 chamber was positioned perpendicular to the beam axis. The entire set of deliveries was repeated, once with the chamber aligned to x‐axis, the second time aligned to y‐axis. The averaged OF from these two orientations were presented as the CC01 OF. The stems of the SFD and the PSD were aligned parallel to the central beam axis. The Edge detector was set up with the cross‐mark aligned to the crosshair. A step size of 0.05 cm was used for the profile and PDD scans. Based on scanned profiles, the detectors were repositioned to the radiation central axis instead of the mechanical central axis indicated by the crosshair (the difference was noted to be less than 0.1 cm). For each radiation exposure, 100 MUs were delivered. Measurements were averaged over a minimum of three repeated deliveries. Measurement reproducibility was within 0.2% (1 SD) for all detectors at each setup. All measurements were repeated three times with independent setups.

In the present study, a $3\times 3\;{\text{cm}}^{2}$ was chosen as an intermediate field size to normalize the diode and CC01 ion chamber readings against the CC04 ion chamber measurements following the “daisy‐chain” approach in Eq. (3):

$$OF_{\text{detector}}=\frac{R_{\text{detector}\_FS}}{R_{\text{detector}\_3\times 3}}\times \frac{R_{CC04\_3\times 3}}{R_{CC04\_10\times 10}}$$

This “daisy chain” normalization approach is recommended to minimize the effect of the diode energy variation on the OF.^(27)^ The CC01 ion chamber is known to exhibit an energy response given the presence of a steel central electrode.^12^, ^14^ Therefore the same normalization procedure was applied to the CC01 ion chamber readings.

The uncertainty associated with the OF presented in this work was estimated using error propagation from both type A and B uncertainties.^(28)^ Type A uncertainty was estimated from the repeated measurements taken for each detector, characterizing the repeatability of MU delivery and charge collection of the detectors.^(20)^ Type B uncertainty was calculated from the uncertainties associated with setting up the SSD (±0.5mm), jaw opening reproducibility (±0.2mm), and positioning the detector (±0.25mm) in three dimensions.

The plastic scintillator dosimetry system employed in this study consists of a polystyrene scintillating fiber with active volume of 1 mm diameter by 3 mm length, an acrylic (PMMA) optic fiber with 1 mm diameter core, a photodiode with two channel output, and a SuperMAX electrometer (Standard Imaging) for online Cerenkov radiation correction. Prior to measurement, calibration of the PSD was performed in solid water at isocenter and 2 cm depth, using 6XFFF beam on the Edge linac. The system was calibrated for Cerenkov radiation by evaluating the different scintillating light outputs in $40\times 40\;{\text{cm}}^{2}$, with maximum versus minimum length of optic fibers in field. The dual‐channel method^(29)^ derives the gain and Cerenkov light ratio (CLR) as below:^(30)^

$$CLR=\frac{SC1_{\text{max}40}-SC1_{\text{min}40}}{SC2_{\text{max}40}-SC2_{\text{min}40}}$$

$$Gain=\frac{1}{\left(SC1_{\text{min}40}-SC2_{\text{min}40}\times CLR\right)}$$

where ${\text{SC}1}_{\text{max}40}$ is the scintillator channel 1 measurement, maximum fiber configuration in $40\times 40\;{\text{cm}}^{2}$; ${\text{SC}1}_{\text{min}40}$ is the scintillator channel 1 measurement, minimum fiber configuration in $40\times 40\;{\text{cm}}^{2}$; ${\text{SC}2}_{\text{max}40}$ is the scintillator channel 2 measurement, maximum fiber configuration in $40\times 40\;{\text{cm}}^{2}$; and ${\text{SC}2}_{\text{min}40}$ is the scintillator channel 2 measurement, minimum fiber configuration in $40\times 40\;{\text{cm}}^{2}$.

The calibrated gain and CLR were subsequently used to autocorrect the OF readings. Since the PSD is energy independent, no daisy‐chain was required.

Type B uncertainty for the PSD was calculated as the root mean square of the total square uncertainties associated with SSD setup (±0.5mm), detector positioning (±0.25mm), jaw opening reproducibility (±0.2mm), and the calibration procedure.

### B. Film measurement

Slabs of solid water ($30\times 30\times 20\;{\text{cm}}^{3}$, Gammex, Inc., Middleton, WI) were aligned to the central axis at 100 cm SSD. Gafchromic EBT3 films were placed at 10 cm depth in between solid water slabs. For each exposure, two films were stacked together to eliminate random film imperfections. Each field size was exposed three times. A pinpoint ion chamber was placed 6 cm downstream from the films in solid water. Ion chamber readings were taken at the same time of film exposure to ensure machine output constancy.

**Figure 1:**
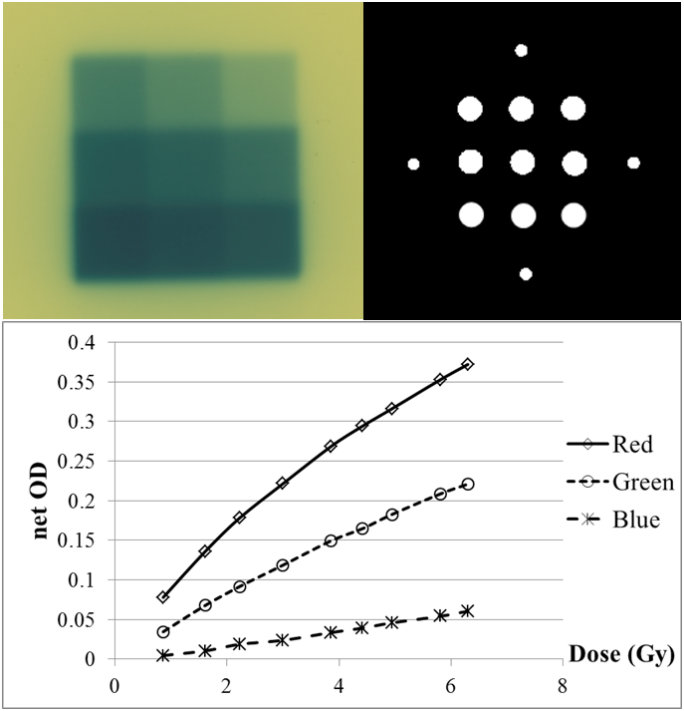
(top left) Calibration film in a 9 square pattern with ranging doses; (top right) the mask used to extract pixel values from the center of each square, as well as four circles outside for background readings; (bottom) the dose versus net OD plots for blue, red, and green channel.

An in‐house film dosimetry protocol was developed to convert optical density to dose. Calibration films were irradiated in a nine‐($2\times 2\;{\text{cm}}^{2}$) square dose pattern, with doses ranging from 0.9 Gy to 6.4 Gy (Fig. 1). The optical densities sampled from the squares were paired with their corresponding doses calculated using the Eclipse TPS (Varian Medical Systems). A calibration curve was generated for each color channel using cubic polynomial least squares fitting. In‐house cross comparison of film dose with chamber measurement revealed less than 2% uncertainty in the red channel for doses less than 10 Gy. Thus the red channel was used for absolute dose film dosimetry. In order to utilize the optimum dose range for the red channel, 800 MUs were delivered for each field size to obtain doses of approximately 5 Gy to the film.

On each film, a $1\times 1{\text{mm}}^{2}$ region of interest (ROI) was chosen on the central axis. Dose from every pixel in the ROI was averaged to generate the central axis dose reading. $1\times 1{\text{mm}}^{2}$ ROI provided a large enough sample of pixels, while staying within the high‐dose region for each field. Output factors were calculated by normalizing the small field dose to the $10\times 10\;{\text{cm}}^{2}$ reference field dose. In addition to output factors, cross‐plane and in‐plane profiles were also extracted. All doses were reported in absolute terms.

The standard deviation of optical densities at the center of each field was computed as the type A uncertainty for film measurements. The type B uncertainty was estimated using error propagation, incorporating the uncertainties in film calibration, SSD set up (±0.5mm), and jaw opening reproducibility (±0.2mm).

### C. Monte Carlo (MC) simulation

Radiation interactions within the linac treatment head are provided in the form of a phase space (PS) file (distributed by the vendor,^(25)^ Varian Medical Systems), containing information related to the position, energy, directionality and type of every particle, and located in a plane perpendicular to the beam central axis, just above the field defining jaws. For the OF calculations, the PS file was used as input, and particle transport within the field defining jaws, specified at relevant field sizes, were simulated using the BEAMnrc code system.^(31)^ From these simulations, secondary PS files were produce at a location below the jaws, and above the patient. Particle interactions, starting with the secondary PS files, were subsequently scored using the DOSXYZnrc program to calculate three‐dimensional dose distributions in a virtual water phantom ($20\times 20\times 20\;{\text{cm}}^{3}$).^18^, ^19^ The voxel resolution of the phantom was set to 1 mm to minimize volume averaging effects. The 3D dose distributions were calculated for a single AP field at 16 jaw‐defined small fields and $10\times 10\;{\text{cm}}^{2}$ reference field. Photon and electron cutoff energies were 350 keV and 5 keV, respectively. The calculated 3D doses were normalized to the maximum dose at the reference geometry, $10\times 10\;{\text{cm}}^{2}$ at 10 cm depth. A large enough number of histories was employed to reduce the statistical uncertainty to ∼0.5% at the maximum dose voxel. In addition to OFs’ PDDs, profiles were extracted and compared against measurements.

## III. RESULTS

Table 2 summarizes the OFs measured with all five detectors. MC calculations for corresponding field sizes are also listed. Relative to the benchmark measurement (performed with the plastic scintillator), the SFD overestimated output factors by 2.5%±0.4% (max.=3.3% for $0.5\times 1\;{\text{cm}}^{2}$ rectangular field). The Edge detector also overestimated the OFs by an average of 4.2%±0.9% (max.=6.0% for $1\times 1\;{\text{cm}}^{2}$ square field). Gafchromic film showed the best agreement with the PSD, with differences within 0.6%±1.8%. MC calculations agreed with the PSD measurements within 1.2%±1.5%. Not unexpectedly, the CC01 measured much lower OFs compared to the other detectors, especially at field sizes smaller than 1 cm, where the differences were up to 11.7% relative to the PSD. The average interdetector difference was 6.6%±3.5% across the 16 fields, with a maximum difference of 14.2% at $0.5\times 0.5\;{\text{cm}}^{2}$ between the CC01 ion chamber and the Edge diode detector. The two diode readings exhibited an average difference of 1.7%±0.7%, with the Edge detector OFs consistently higher than that of SFDs.

Correction factors were calculated for CC01, SFD, Edge detector, film, and Monte Carlo, using PSD as reference. The results are listed in Table 3.

Table 4 shows the collimator exchange (CE) effects quantified in percentages (see Eq. (1)) for all jaw combinations. The CE effect was most prominent at the smallest field sizes studied, with either jaw set to 0.5 cm. For example, the OF for $2\times 0.5\;{\text{cm}}^{2}$ is smaller than that of $0.5\times 2\;{\text{cm}}^{2}$ by 10.6%, according to PSD measurements. The averaged collimator exchange effect was 4.2%±4.5% across all detectors and field sizes.

**Table 2:** Output factors from five detectors and Monte Carlo simulation. Corresponding uncertainties at the 68% level (1 SD) are listed in parenthesis

*Output Factor*												
	*CC01*				*SFD*				*Edge*			
*X\Y(cm)*	*0.5*	*1*	*1.5*	*2*	*0.5*	*1*	*1.5*	*2*	*0.5*	*1*	*1.5*	*2*
0.5	0.40 (0.02)	0.53 (0.01)	0.55 (0.01)	0.56 (0.01)	0.47 (0.00)	0.60 (0.01)	0.61 (0.01)	0.62 (0.01)	0.47 (0.00)	0.60 (0.01)	0.62 (0.01)	0.63 (0.01)
1	0.52 (0.02)	0.70 (0.00)	0.73 (0.00)	0.74 (0.00)	0.54 (0.01)	0.70 (0.00)	0.74 (0.00)	0.75 (0.00)	0.56 (0.00)	0.73 (0.00)	0.75 (0.00)	0.76 (0.00)
1.5	0.54 (0.02)	0.73 (0.00)	0.77 (0.00)	0.78 (0.00)	0.55 (0.01)	0.73 (0.00)	0.76 (0.00)	0.78 (0.00)	0.57 (0.00)	0.75 (0.00)	0.78 (0.00)	0.79 (0.00)
2	0.55 (0.03)	0.74 (0.00)	0.78 (0.00)	0.80 (0.01)	0.56 (0.01)	0.74 (0.00)	0.78 (0.00)	0.79 (0.00)	0.57 (0.00)	0.75 (0.00)	0.79 (0.00)	0.80 (0.00)
	*Film*				*Monte Carlo*				*PSD*			
*X\Y(cm)*	*0.5*	*1*	*1.5*	*2*	*0.5*	*1*	*1.5*	*2*	*0.5*	*1*	*1.5*	*2*
0.5	0.45 (0.02)	0.57 (0.03)	0.59 (0.03)	0.59 (0.02)	0.45 (0.01)	0.58 (0.01)	0.60 (0.01)	0.60 (0.01)	0.46 (0.01)	0.58 (0.00)	0.60 (0.00)	0.61 (0.00)
1	0.53 (0.02)	0.69 (0.02)	0.72 (0.02)	0.74 (0.02)	0.54 (0.01)	0.71 (0.01)	0.72 (0.01)	0.73 (0.01)	0.53 (0.01)	0.69 (0.00)	0.72 (0.00)	0.73 (0.00)
1.5	0.54 (0.03)	0.73 (0.08)	0.76 (0.02)	0.78 (0.02)	0.55 (0.01)	0.73 (0.01)	0.76 (0.01)	0.77 (0.01)	0.54 (0.01)	0.71 (0.01)	0.75 (0.00)	0.76 (0.00)
2	0.55 (0.02)	0.74 (0.02)	0.78 (0.02)	0.79 (0.02)	0.56 (0.01)	0.74 (0.01)	0.78 (0.01)	0.78 (0.01)	0.55 (0.01)	0.72 (0.00)	0.76 (0.00)	0.78 (0.00)

**Table 3:** Ratio of output factors between the PSD and other detectors (i.e., detector‐specific correction factor $k_{Q_{\text{clin},Q_{\text{msr}}}}^{f_{\text{clin},f_{\text{msr}}}}$) for the CC01, SFD, and Edge. Ratios for film and Monte Carlo are also reported. Corresponding uncertainties at the 68% level (1 SD) are listed in parenthesis

$k_{Q_{\text{clin},Q_{\text{msr}}}}^{f_{\text{clin},f_{\text{msr}}}}$			
	*CC01*				*SFD*				*Edge*			
*X\Y(cm)*	*0.5*	*1*	*1.5*	*2*	*0.5*	*1*	*1.5*	*2*	*0.5*	*1*	*1.5*	*2*
0.5	1.13 (0.02)	1.09 (0.01)	1.08 (0.01)	1.08 (0.01)	0.98 (0.01)	0.97 (0.01)	0.98 (0.01)	0.97 (0.01)	0.98 (0.01)	0.96 (0.01)	0.97 (0.01)	0.97 (0.01)
1	1.02 (0.02)	0.98 (0.00)	0.98 (0.00)	0.98 (0.00)	0.98 (0.01)	0.97 (0.00)	0.97 (0.00)	0.97 (0.00)	0.95 (0.01)	0.94 (0.00)	0.95 (0.00)	0.95 (0.00)
1.5	1.01 (0.02)	0.97 (0.01)	0.97 (0.00)	0.97 (0.00)	0.98 (0.01)	0.97 (0.01)	0.98 (0.00)	0.98 (0.00)	0.96 (0.01)	0.95 (0.01)	0.96 (0.00)	0.96 (0.00)
2	1.00 (0.03)	0.97 (0.01)	0.97 (0.01)	0.98 (0.01)	0.98 (0.01)	0.97 (0.01)	0.98 (0.00)	0.98 (0.00)	0.96 (0.01)	0.95 (0.01)	0.96 (0.00)	0.97 (0.00)
	*Film*				*Monte Carlo*				*PSD*			
*X\Y(cm)*	*0.5*	*1*	*1.5*	*2*	*0.5*	*1*	*1.5*	*2*	*0.5*	*1*	*1.5*	*2*
0.5	1.02 (0.02)	1.01 (0.03)	1.02 (0.03)	1.02 (0.02)	1.02 (0.01)	1.00 (0.01)	1.01 (0.01)	1.00 (0.01)	References			
1	1.00 (0.02)	0.99 (0.02)	0.99 (0.02)	0.98 (0.02)	0.98 (0.01)	0.97 (0.01)	0.99 (0.01)	1.00 (0.01)				
1.5	1.00 (0.03)	0.97 (0.08)	0.98 (0.02)	0.98 (0.02)	0.99 (0.01)	0.97 (0.01)	0.98 (0.01)	0.99 (0.01)				
2	1.00 (0.02)	0.97 (0.02)	0.97 (0.02)	0.99 (0.02)	0.98 (0.01)	0.97 (0.01)	0.98 (0.01)	1.00 (0.01)				

**Table 4:** Collimator exchange effect (%) for nonsquare fields by five detectors and Monte Carlo, calculated using Eq. (1)

*X,Y (cm)*	*Jaw Ratio*	*CC01*	*SFD*	*Edge Detector*	*Film*	*Monte Carlo*	*PSD*
0.5, 1	2	2.1%	10.3%	8.8%	7.9%	7.6%	9.3%
0.5, 1.5	3	2.7%	10.8%	9.7%	8.3%	8.3%	10.2%
0.5, 2	4	2.5%	11.1%	9.7%	8.3%	8.3%	10.6%
1, 1.5	1.5	0.7%	1.1%	0.9%	‐0.5%	‐0.5%	1.2%
1, 2	2	0.6%	1.3%	1.1%	‐0.3%	‐1.9%	1.2%
1.5, 2	1.3	0.0%	0.2%	0.2%	‐0.4%	‐0.8%	0.2%

Monte Carlo (MC) simulation was performed for all 16 field sizes. The MC code was first validated against the square field measurements. There is currently no commercial scanning tank compatible with the PSD,^(32)^ as a two‐channel output system is required to account for the Cerenkov light ratio correction with varying fiber lengths in the field. The Edge diode detector was thus utilized for PDD comparison against MC calculations, while EBT3 film measurements were used for the absolute dose profile comparisons.

**Figure 2:**
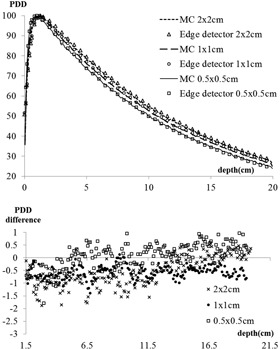
(top) Monte Carlo simulated and Edge detector measured 6XFFF PDD curves for three small fields, $2\times 2\;{\text{cm}}^{2},1\times 1\;{\text{cm}}^{2}$, and $0.5\times 0.5\;{\text{cm}}^{2}$; (bottom) difference map for PDDs beyond the buildup region (1.5 cm) for the three small fields.

At the standard calibration field size of $10\times 10\;{\text{cm}}^{2}$, the average PDD difference between MC and Edge detector was 0.7%±2.0%. Beyond dmax, the average difference was 0.8%±0.5%. At small fields (Fig. 2) beyond dmax, the average PDD differences between MC and Edge detector were 0.6%±0.6%,0.6%±0.3%, and 0.1%±0.5% in $2\times 2\;{\text{cm}}^{2},1\times 1\;{\text{cm}}^{2}$, and $0.5\times 0.5\;{\text{cm}}^{2}$ fields, respectively. The buildup regions agreed within 1 mm.

**Figure 3:**
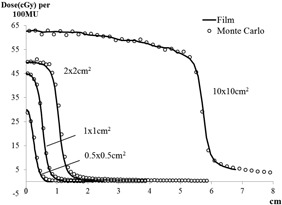
Half profiles simulated by Monte Carlo, and measured by film, for field sizes $10\times 10\;{\text{cm}}^{2},2\times 2\;{\text{cm}}^{2},1\times 1\;{\text{cm}}^{2}$, and $0.5\times 0.5\;{\text{cm}}^{2}$.

For cross‐profiles (Fig. 3), the absolute dose difference was computed between MC and EBT3 film measurements. At $10\times 10\;{\text{cm}}^{2}$, the high‐dose region (>90% of central axis (CAX) dose) agreed within 0.6±0.9cGy; the low‐dose region (<10% of CAX dose) agreed within 0.6±0.6cGy; the average distance‐to‐agreement (DTA) at penumbra (10–90% of CAX dose) was 0.13 cm. Similarly at small fields, the high dose differences were 0.9±0.6cGy ($2\times 2\;{\text{cm}}^{2}$), 0.4±0.3cGy ($1\times 1\;{\text{cm}}^{2}$), and 0.1±1.0cGy ($0.5\times 0.5\;{\text{cm}}^{2}$). The low dose differences were 1.2±0.2cGy ($2\times 2\;{\text{cm}}^{2}$), 0.9±0.2cGy ($1\times 1\;{\text{cm}}^{2}$), and 1.6±0.5cGy ($0.5\times 0.5\;{\text{cm}}^{2}$). The DTA at penumbra were 0.10 cm, 0.08 cm, and 0.002 cm for $2\times 2\;{\text{cm}}^{2},1\times 1\;{\text{cm}}^{2}$, and $0.5\times 0.5\;{\text{cm}}^{2}$ fields, respectively.

## IV. DISCUSSION

The topic of detector‐specific correction factors for small fields has been extensively investigated in the literature.^(12,14,15,17–21)^ However, the majority of these studies have focused on square fields,^12^, ^14^, ^17^, ^21^ and very few include measurements of rectangular fields in FFF beams.^(18)^ Unlike square fields, output factors in rectangular fields are susceptible to the collimator exchange effect. There are two major components contributing to this collimator exchange effect: jaw backscatter and penumbra.

Depending on the size defined by the upper Y jaw, different amounts of backscattered photons are generated.^33^, ^34^ A smaller upper jaw (Y) opening induces more backscatter into the monitor chamber, which subsequently results in fewer MUs delivered, producing a smaller OF. For instance, the smaller Y opening for $1\times 0.5\;{\text{cm}}^{2}$ (XxY) contributed to a smaller OF (0.53), compared to the OF (0.58) for the opposite jaw setting $0.5\times 1\;{\text{cm}}^{2}$ (XxY).

**Figure 4:**
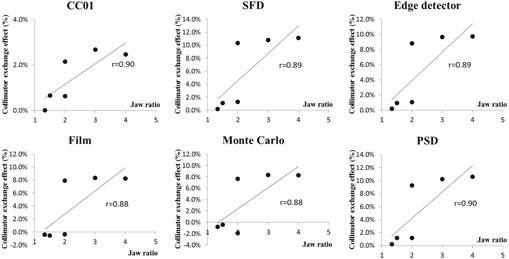
Collimator exchange effect (Eq. (1)) plotted against jaw ratios (Y/X). The correlation coefficient (*r*) was calculated for each detector.

**Figure 5:**
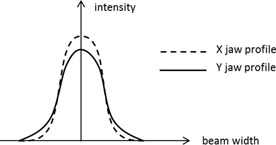
Illustration of the penumbral effect at very small fields (<1cm). Intensity profiles plotted for the same size aperture formed by XxY vs. YxX jaws. The X (lower) jaws form the smallest aperture.

In addition to backscatter, the penumbra effect also contributes to CE, as observed in Table 4 and Fig. 4. The penumbra effect refers to the rapid dose falloff at small fields <1cm (i.e., the beam profile is mostly made up of penumbra). Since the X jaw is located closer to the isocenter, it casts a sharper penumbra than the Y jaw. Figure 5 illustrates this effect for profiles of the same aperture, formed by XxY versus YxX jaws. The sharper penumbra casted by the X jaws, when the X jaw forms the smallest aperture appears to contribute to a higher OF.

Another contributing factor is the shape of source. Scott et al.^(35)^ demonstrated that Varian machines have a tilted elliptical shape source. At certain angles, an elongated jaw aperture result in higher OFs than at other angles. Additional measurements were performed with 0°, 45°, and 90° collimator rotations. A small but consistent change (1%–2%) in OF was observed, with the highest reading at 90°. This observation agreed with previous work of Scott et al.^(35)^ and Jaffray et al.,^(36)^ where a spot size slightly larger in the Y direction was reported.

The collimator exchange effect was observed for all detector measurements as well as MC simulations. CC01 presented the lowest CE effect, due to the volume averaging effect. Correlations between jaw ratio and the CE effect were computed and are plotted in Fig. 4. CE effects were found to correlate with field elongation for all detectors, with the correlation coefficient r>0.8. The more elongated the aperture, the larger the CE effect. All detectors achieved similar correlation coefficient, ranging from 0.88 to 0.90. Two distinct groups of CE can be observed in all the plots. The three data points with the largest CE effects belonged to fields with either jaw set at 0.5 cm. This was due to the diminishing source occlusion as the field size exceeds 0.5 cm, as demonstrated by Kumar et al.^(8)^ Francescon et al.^(17)^ studied small rectangular field OFs in two linacs: Synergy (Elekta, Stockholm, Sweden) and Primus (Siemens, Munich, Germany). Similar CE effects were observed on these linacs as CE observed in this work (Varian Edge). The presence of a large CE effect at small field sizes implies that this effect cannot be ignored for small field dosimetry, especially for treatment planning of small, elongated fields, as routinely used for spine SRS.

The Varian source model uses a collimator backscatter factor (CBSF) calculation, which employs the jaw‐based output factor table in Eclipse to determine the backscatter to the monitor chamber. Currently, Eclipse only takes square field OFs down to $1\times 1\;{\text{cm}}^{2}$. This means the CE effect is ignored in the treatment planning system. Dose calculation for small field consists of three major factors: CBSF, dosimetric leaf gap (DLG), and virtual source size. Literature focused on the DLG because the apertures are formed by the MLCs, and changes in DLG affect the modeling of MLC leaf ends. Jaws were not emphasized in literature as much in the context of small field, because the jaws were rarely used without MLCs. However, jaw output factors directly determine the CBSF, and consequently the MUs delivered. The application of jaw‐tracking feature in TrueBeam‐type machines also calls for accurately modeled jaws. Our results demonstrated up to 11.1% collimator exchange effect at small jaw fields of 0.5–2 cm. The lack of account for discrepancies of such magnitude could lead to noticeable differences in the high‐dose region.

The ion chamber, the standard for large field dosimetry, suffers from volume averaging effect at small field sizes. Lechner et al.^(18)^ reported a similar underestimation (10%) for the CC01 ion chamber at $0.5\times 0.5\;{\text{cm}}^{2}$, which are in reasonable agreement with our results (11.7%) despite the slight differences in experimental setup. Volume averaging effect also contributed to the smallest CE among all detectors.

Diodes overrespond at small fields due to the extra perturbation contributed by the increased electron fluence and higher stopping power in silicon. Authors have reported correction factors $k_{Q_{\text{clin},Q_{\text{msr}}}}^{f_{\text{clin},f_{\text{msr}}}}$ for the SFD and Edge detector diodes at 0.5 cm.^(13,15,18,19,21)^ Our results agree with published data within 2%. Interestingly, the response of the Edge detector was consistently higher than that of the SFD for all field sizes studied. This could be attributed to the additional shielding in the Edge detector, originally designed to absorb low‐energy scatter photons. The brass shielding may have induced additional dose perturbation, and therefore a higher response relative to the unshielded SFD. Dieterich et al.^(14)^ and Francescon et al.^(17)^ both reported similar over‐responses with the Edge detector versus the SFD. The effect of extra shielding was further validated by Lechner et al.,^(18)^ who discovered that the low‐energy scattered photons in flattened beams tend to induce higher response in shielded diodes, such as the Edge detector.

The use of radiochromic film for small field dosimetry is advantageous due to the film high spatial resolution, water equivalence, and convenient two‐dimensional dose display.^(23)^ A number of studies have used Gafchromic EBT films as a reference in small field dosimetry.^21^, ^37^ The film program employed in the present study has been proven robust in terms of absolute dosimetry, and was detailed in a previous publication.^(38)^ In addition to the excellent OF agreement with the PSD (0.6%), profiles obtained by film were further verified against Monte Carlo calculations and found to be in good agreement.

Similar to radiochromic film, the PSD is preferred for small field dosimetry due to independence on energy, dose rate, temperature, and water equivalence. The dose perturbation effect is also minimal with the PSD, considering it has a physical density of 1.0 5 g/cm.^(39)^ One major advantage of PSD over film is its instantaneous readout, which was favorable for clinical dosimetry. In this study, the PSD was verified against MC calculations (average difference of 1.2%), in agreement with Wang and Beddar's study,^(24)^ where differences of up to 1.5% were observed between PSD and MC at 0.5 cm. Differences between the PSD and film were also proven minimal in this study.

## V. CONCLUSIONS

In commissioning a dedicated radiosurgery linac equipped with FFF beam, output factors for 16 small fields were measured and simulated using the Monte Carlo method. Interdetector differences revealed severe underestimation of OFs measured with ion chambers, and moderate overestimation with silicon diodes, relative to a plastic scintillator at the smallest (0.5 cm) field sizes. The CC01 ion chamber should not be used in small field commissioning. The SFD and Edge detector can be used with an understanding of their limitations. The PSD results were verified against EBT3 film measurements and MC calculations and were found to be in good agreement. Detector‐specific correction factors reported for squared fields were in good agreement with the literature.

Among the field sizes studied, a correlation was discovered between field elongation and collimator exchange effect. Caution is required when evaluating dosimetric accuracy at these small elongated field sizes. More research is warranted to fully understand the collimator exchange effect for small rectangular fields.

## COPYRIGHT

This work is licensed under a Creative Commons Attribution 3.0 Unported License.
